# Digoxin promotes anoikis of circulating cancer cells by targeting Na^+^/K^+^-ATPase α3-isoform

**DOI:** 10.1038/s41419-025-07703-z

**Published:** 2025-05-11

**Authors:** Yoshihisa Numata, Takuto Fujii, Chihiro Toda, Tomoyuki Okumura, Takahiro Manabe, Naoya Takeda, Takahiro Shimizu, Yoshiaki Tabuchi, Tsutomu Fujii, Hideki Sakai

**Affiliations:** 1 Department of Surgery and Science, Faculty of Medicine, Academic Assembly, University of Toyama, Toyama, 930-0194 Japan; 2 Department of Pharmaceutical Physiology, Faculty of Pharmaceutical Sciences, University of Toyama, Toyama, 930-0194 Japan; 3 Division of Molecular Genetics Research, Life Science Research Center, University of Toyama, Toyama, 930-0194 Japan

**Keywords:** Metastasis, Gastric cancer, Apoptosis

## Abstract

Circulating cancer cells (CCCs) are closely related to the process of distant metastasis. In early step of the metastasis cascade, CCCs must evade the detachment-induced cell death (anoikis) for their survival. Here, we examined whether Na^+^/K^+^-ATPase α3-isoform (α3NaK) in CCCs contributes to avoidance of anoikis. In CCCs isolated from gastric cancer patients, α3NaK was predominantly localized in the plasma membrane (PM), but it moved to the cytoplasm when the CCCs were attached to culture dishes. The CCCs showed significant expression of integrin α5 but not fibronectin, one of components of the extracellular matrix (ECM). In human gastric cancer MKN45 cells, digoxin (20 and 50 nM), a cardiac glycoside, significantly inhibited the enzyme activity and translocation (from cytoplasm to PM) of α3NaK, while they had no significant effect on ubiquitous Na^+^/K^+^-ATPase α1-isoform (α1NaK) in the PM. The translocation of α3NaK required the loss of ECM components from the cells. Additionally, digoxin significantly enhanced caspase 3/7 activity, as well as the expression of cleaved caspase 3, while reducing the viability of detached (floating) cells. In the MKN45 xenograft mouse model, intraperitoneal administration of digoxin (2 mg/kg/day) significantly decreased the number of CCCs and suppressed their liver metastasis. Our results suggest that α3NaK plays an essential role in the survival of CCCs in gastric cancer, and that digoxin enhances anoikis in detached (metastatic) gastric cancer cells by inhibiting the α3NaK translocation from cytoplasm to PM, thereby reducing CCCs. Targeting α3NaK may be a promising therapeutic strategy against CCC survival.

## Introduction

Gastric cancer is the fifth most frequent cancer and the third leading cause of cancer-related deaths worldwide [1]. Despite recent advancements in diagnosis and comprehensive therapy which led to an improvement in 5-year survival, approximately 50% of gastric cancer patients suffer from cancer recurrence or metastasis after curative resection [2, 3]. Distant metastasis of gastric cancer most commonly occurs in the liver, which is the leading cause of gastric cancer-related mortality, with a 5-year survival rate of less than 5% [4, 5].

Circulating cancer cells (CCCs) are cancer cells detached from the primary tumor and circulate in the bloodstream. CCCs are frequently found in various solid tumor types and are considered precursors to metastasis in cancers [6]. Many studies have shown that the number of CCCs can be correlated with disease progression and poor prognosis in various cancers, including gastric cancer [7–14]. Thus, reducing the number of CCCs is crucial for developing effective and innovative treatment strategies to prevent cancer metastasis.

Anoikis is cell death when cells are detached from the extracellular matrix (ECM), a three-dimensional network of macromolecules [15]. In the early step of cancer metastasis, CCCs acquire resistance to anoikis, allowing them to survive during circulating in the bloodstream [16–18]. Therefore, the acquisition of anoikis resistance in CCCs is considered as one of key events for promoting cancer recurrence and metastasis, and overcoming anoikis resistance can reduce CCC survival and prevent metastasis [16, 17].

Na^+^/K^+^-ATPase α1-isoform (α1NaK) is expressed in the plasma membrane (PM) of almost all cells and contributes to maintaining the resting membrane potential and transmembrane gradients for Na^+^ and K^+^ [19, 20]. On the other hand, Na^+^/K^+^-ATPase α3-isoform (α3NaK) is highly expressed in the PM of neuronal cells, playing a crucial role in restoring membrane potential after depolarization and in maintaining neuronal excitability [21]. We reported recently that α3NaK is abnormally expressed in intracellular vesicles of various human cancer cell lines under the cell-attached condition, and that the intracellular α3NaK is translocated to the PM upon cancer cell detachment in vitro [22]. This finding may explain one of the mechanisms for the survival of detached cancer cells.

Interestingly, previous epidemiological studies showed that treatment with cardiac glycosides, such as digitoxin and digoxin, prevents cancer recurrence and improves survival in cancer patients [23]. Thus, Na^+^/K^+^-ATPase, a target of cardiac glycosides, has been considered as a potent molecule with clinical benefit in cancer treatment [24–29].

In the present study, we examined whether α3NaK contributes to the survival of CCCs and whether digoxin is effective for attenuating the survival of CCCs by using mouse models in vivo.

## Materials and methods

### Materials

Digoxin, human fibronectin, human collagen type IV, human laminin, and anti-flotillin-2 antibody were obtained from Sigma-Aldrich. Fetal bovine serum (FBS) was from Nichirei bioscience or Sigma-Aldrich. Anti-α1NaK and anti-α3NaK antibodies were from Santa Cruz Technology. Anti-fibronectin and anti-integrin α5 antibodies, and HRP-conjugated anti-rat IgG were from Proteintech. Anti-cleaved caspase-3 antibody was from Cell signaling technology. Human and mouse CD45 recombinant proteins were from Fujifilm Wako. DAPI was from Dojindo Laboratories. Anti-EpCAM antibody was from Miltenyi Biotec. HRP-conjugated anti-mouse and rabbit IgGs were from Millipore. Alexa Fluor 488- or 568-conjugated antibodies were from Abcam.

### Patients

Gastric cancer tissues and blood samples were collected from Japanese patients in accordance with the recommendations of the Declaration of Helsinki and with ethics committee approval of the University of Toyama (Approval No. 22-45, 29-85, and R2023238). Informed consent was obtained from all patients at Toyama University Hospital. Age (years) and sex (M or F) of the patient, histological type (grading), and stage of the carcinoma according to TNM classification (I, II, III or IV) are as follows: No. 1 (87, M, tubular (mod), stage IIIb), No. 2 (83, M, tubular (mod), stage IV), No. 3 (69, M, tubular (por), stage IV), No. 4 (64, M, tubular (mod), stage IIA), No. 5 (63, M, tubular (mod), stage IIA), No. 6 (57, M, papillary adenocarcinoma, stage IA), No. 7 (68, M, tubular (well), stage II), and No. 8 (70, M, tubular (poor), stage IIIA).

### Isolation of CCCs from gastric cancer patients

**Fig. 1:**
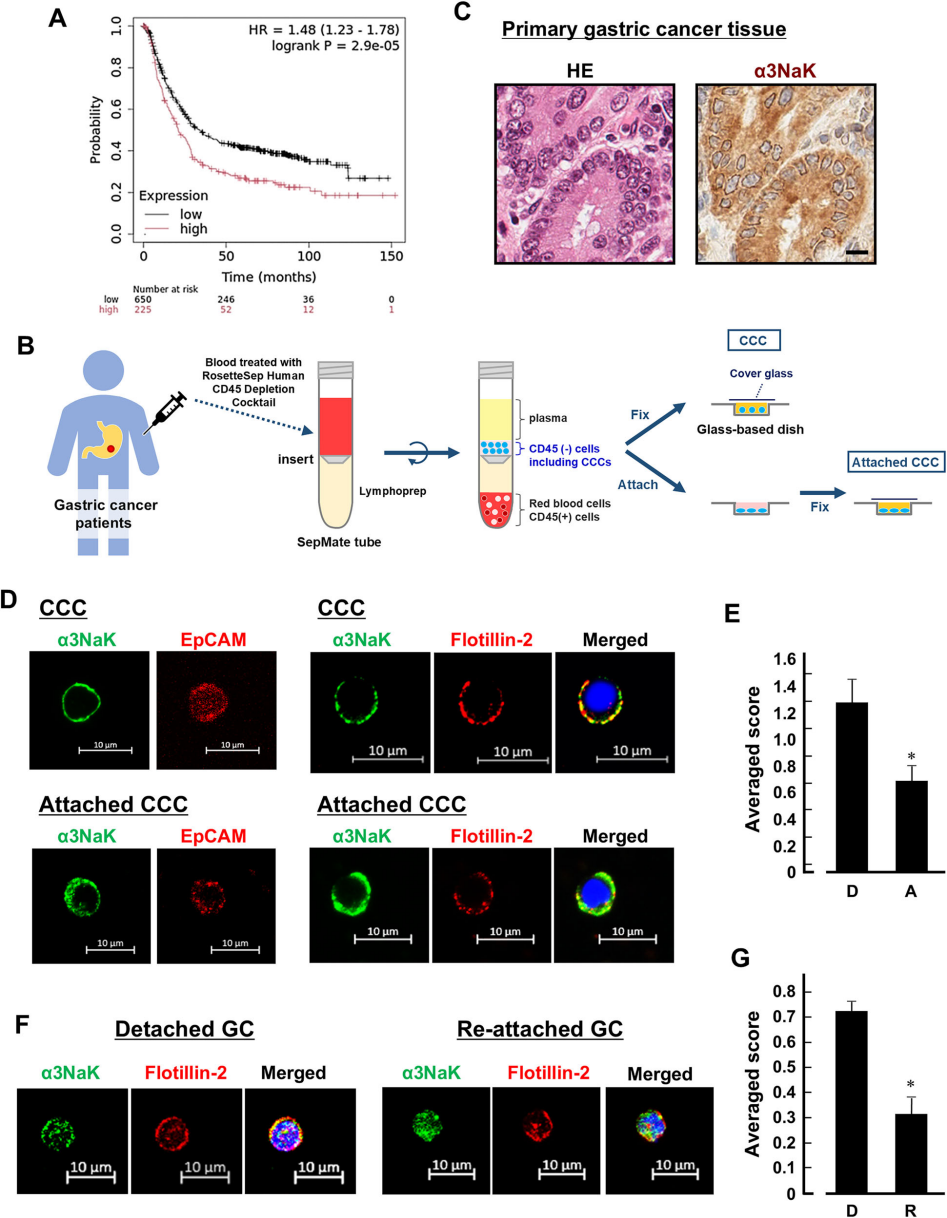
Expression of α3NaK in CCCs of patients with gastric cancer. **A** Kaplan-Meier Plotter analysis of α3NaK in gastric cancer. **B** A schematic overview of the experimental procedure for isolation of CCCs from patients with gastric cancer. CCCs were isolated and enriched using the RosetteSep™ Human CD45 Depletion Cocktail and Lymphoprep, which depletes white and red blood cells by density centrifugation with tetrameric antibody complexes recognizing CD45. The CD45(-) cells, including CCCs, were fixed with 4% paraformaldehyde on the glass-based dish. When indicated, the cells were attached to a poly-L-lysine-coated dish before fixation. **C** Hematoxylin and eosin (HE) staining and immunohistochemistry using anti-α3NaK antibody were performed in primary gastric cancer tissues. Scale bar, 10 μm. **D** Immunocytochemistry was performed using antibodies for α3NaK (green), EpCAM (a marker for CCCs; red), and flotillin-2 (a marker for PM; red) in CCCs and attached CCCs of gastric patients. Scale bars, 10 μm. **E** The distribution of α3NaK in detached (D) and attached (A) CCCs were scored as 0 (predominantly at cytoplasm), 1 (both at cytoplasm and PM), and 2 (predominantly at PM) in (**D)**. Averaged scores in detached (*n* = 18) and attached (*n* = 29) CCCs were shown. *p < 0.05. **F** Immunocytochemistry was performed using antibodies for α3NaK (green), EpCAM (red), and flotillin-2 (red) in detached and re-attached gastric cancer cells (GC) obtained by enzyme digestion of primary cancer tissue. Scale bars, 10 μm. **G** The distribution of α3NaK in detached (D) and re-attached (R) gastric cancer cells obtained by enzyme digestion of primary cancer tissue were scored as 0 (predominantly at cytoplasm), 1 (both at cytoplasm and PM), and 2 (predominantly at PM) in (**F)**. Averaged scores in detached (*n* = 64) and re-attached (*n* = 90) cells were shown. *p < 0.05.

Five milliliters of blood were collected preoperatively from gastric cancer patients (Patients No. 1-5). The blood was mixed with 250 µl RosetteSep Human CD45 Depletion Cocktail (StemCell Technologies) and incubated for 10 min at room temperature. Subsequently, 5 ml PBS containing 2% FBS was added to the mixture and transferred to a Sepmate tube (StemCell Technologies) containing 3.5 ml Lymphoprep (StemCell Technologies) and centrifuged at 1,200 × g for 15 min. The supernatant was centrifuged at 300 × g for 10 min. For hemolysis, the pellet was incubated with 1 ml ammonium chloride on ice for 15 min. The cell pellet was fixed with 4% paraformaldehyde. For attachment of CCCs, the cell pellet was plated on the collagen-coated glass-based dish for 1 h and then fixed (Fig. 1B).

### Enzyme digestion of human gastric cancer tissues

Gastric cancer tissues were digested in MEM containing 10% FBS, 200 units/ml collagenase (Fujifilm Wako), and 667 tyrosine units/ml actinase E (Kaken Pharmaceutical) for 30 min at 37 °C. The solution was centrifuged at 200 × g for 5 min, and the pellet was fixed with 4% paraformaldehyde. For cell re-attachment of the cells, the digested samples were plated onto a collagen-coated glass-based dish for 1 h and then fixed.

### Cell culture

Human gastric cancer MKN45 cells stably expressing luciferase (JCRB Cell Bank; JCRB1379) were maintained in RPMI1640 (Nacalai or Fujifilm Wako) supplemented with 100 units/ml penicillin, 100 μg/ml streptomycin, and 10% FBS. The medium for culturing MKN45 cells under 120 mM Na^+^ and 20 mM Na^+^ conditions was prepared based on the composition of RPMI1640 by supplementing it with MEM vitamin solution (Fujifilm Wako), MEM essential amino acid solution (Fujifilm Wako), MEM non-essential amino acid solution (Fujifilm Wako), 5 mM KCl, 0.6 mM Ca(NO_3_)_2_, 0.4 mM MgSO_4_, 5 mM Na_2_HPO_4_, 1.1 mM D-glucose, 3.25 µM glutathione, 100 units/ml penicillin, 100 μg/ml streptomycin, 2% FBS, HEPES-Tris (pH 7.4), and 110 mM NaCl (for 120 mM-Na^+^ medium) or 10 mM NaCl plus 100 mM NMDG-Cl (for 20 mM-Na^+^ medium).

### Experimental animals

Male BALB/c-nu/nu mice (Cg-Foxn1nu/CrlCrlj, 5 weeks old; NINOX Labo) were housed under controlled conditions of temperature (22 ± 2 °C) and under a 12-h artificial light/dark cycle. The mice had access to food and water *ad libitum*. All animal experiments were conducted in accordance with the Guidelines for the Care and Use of Laboratory Animals at the University of Toyama and were approved by the Animal Experimentation Committee (Approval No. A2020UH-4). Animals were randomly allocated to experimental groups and were euthanized by cervical dislocation under 4% isoflurane inhalation anesthesia if tumor diameter exceeded 20 mm due to tumor growth. Sample processing was conducted under blinded conditions to ensure objective data analysis.

### MKN45 xenografts in nude mice

Six-week-old male nude mice (BALB/c-nu/nu) were anesthetized by intraperitoneal administration of a triad of anesthetics (0.75 mg/kg medetomidine, 4 mg/kg midazolam, and 5 mg/kg butorphanol). MKN45 cells (1.0 × 10^6^ cells in 50 µl PBS) were injected into two sites on the serosal side of the stomach as previously reported [30]. The mice were maintained for seven weeks. Digoxin (2 mg/kg/day) or PBS was intraperitoneally administered for 2 weeks. Blood was then collected, and the stomach and liver were removed. Tumor weight was estimated as follows: total weight of the stomach including cancer tissues was measured, and then, 200 mg (average weight of isolated stomachs from normal mice) was subtracted from the total weight. Liver metastasis was assessed by counting the number of metastases per mouse. The liver was also incubated with Vivo Glo luciferin (300 µg/ml), and the luminescence was measured using a Clairvivo OPT (Shimadzu).

On the other hand, mice were implanted with MKN45 cells (1.0 × 10^7^ cells in 200 µl PBS) in two subcutaneous sites on the back. The mice were then maintained for eight weeks. Digoxin (2 mg/kg/day) or PBS was administered intraperitoneally for 1 week. Blood was collected from the heart, and the subcutaneous tumors were removed.

### Isolation of CCCs from mouse bloods

Mouse blood was incubated with ammonium chloride solution on ice for 15 min to induce hemolysis, and then incubated with anti-CD45 antibody for 10 min at 4 °C. RapidSpheres containing magnetic beads were then added and incubated for 5 min at 4 °C. The plate was placed on a magnet, and the supernatant was transferred to an adjacent well and incubated five times for 30 s. The samples were immunostained with EpCAM and the EpCAM-positive cells were counted.

### Measurement of ATP-hydrolyzing activity of Na^+^/K^+^-ATPase

ATP-hydrolyzing activity of Na^+^/K^+^-ATPase in membrane fractions (30 μg of protein) of MKN45 cells and human gastric cancer tissues (Patients No. 6-8) were measured in either a 20 mM-Na^+^ or a 120 mM-Na^+^ solution as previously reported [29]. Na^+^/K^+^-ATPase activity was calculated as the difference between activities in the presence and absence of 100 μM digoxin.

### Cell biotinylation

MKN45 cells were pretreated with digoxin or dimethyl sulfoxide for 1 h, then harvested with 0.25% trypsin plus EDTA and incubated for 30 min at 37 °C in culture medium containing digoxin or dimethyl sulfoxide. The detached cells were used in the biotinylation assay as previously reported [22]. Biotinylated samples were detected by Western blotting.

### Western blotting

Western blotting was conducted as described previously [31]. For primary antibodies, Anti-α3NaK (1:5,000), anti-α1NaK (1:5,000), anti-integrin α5 (1:1,000), anti-fibronectin (1:1,000), anti-human CD45 (1:1,000), anti-mouse CD45 (1:1,000), and anti-cleaved caspase 3 (1:1,000) antibodies were incubated for 15 h at 4 °C. HRP-conjugated anti-mouse, rabbit, or rat IgG was used as the secondary antibody at a 1:5,000 dilution for 1 h at room temperature. Signals were visualized using LAS-4000 (FujiFilm).

### Immunocytochemistry

Detached (floating) MKN45 cells and CCCs were fixed with 4% paraformaldehyde and plated on poly-L-lysine-coated glass-based dishes. For attached MKN45 cells and CCCs, cells were plated on type 1 collagen-coated glass-based dishes for 1 h before fixation. Immunocytochemistry was performed as previously reported [22]. The cells were incubated with the anti-α3NaK (1:100), anti-flotillin-2 (1:100), anti-integrin α5 (1:100), anti-fibronectin (1:100), and anti-EpCAM (1:100) antibodies overnight at 4°C and then with the Alexa Fluor-conjugated anti-rabbit and mouse IgG antibodies (Abcam; 1:100) for 1 h at room temperature. DNA was visualized using DAPI (1:1,000). Immunofluorescence images were acquired using a Zeiss LSM 780.

### Immunohistochemistry

Immunohistochemistry was performed as described previously [22]. Briefly, formalin-fixed and paraffin-embedded tissues were cut into 4-μm-thick sections. The sections were incubated with anti-α3NaK antibody (1:200) for 15 h and then incubated with the EnVision+ dual link system (DAKO) for 30 min at room temperature. The reaction products were visualized with DAB+ (DAKO), and the nuclei were lightly counterstained with Mayer’s hematoxylin.

### Caspase 3/7 assay

MKN45 cells were pretreated with digoxin or dimethyl sulfoxide for 1 h, then harvested with 10 mM EDTA and cultured in suspension using a magnetic stirrer for 48 h at 37 °C in the culture medium containing digoxin or dimethyl sulfoxide. The cells (4 × 10^4^ cells) were mixed with Caspase-Glo 3/7 assay reagent (Promega) and incubated for 1 h at room temperature. The luminescence was measured using a Filter Max F5.

### Cell viability

MKN45 cells were pretreated with digoxin or dimethyl sulfoxide for 1 h. The cells were then harvested with 10 mM EDTA and cultured in suspension using a magnetic stirrer for 48 h at 37 °C in the culture medium containing digoxin or dimethyl sulfoxide. The cells (2 × 10^5^ cells) were mixed with MTT reagents (Cayman) and incubated for 3 h at 37 °C and then centrifuged at 300 × g for 5 min. The pellets were dissolved in a crystal-dissolving solution, and the absorbance was measured at 570 nm.

### Statistical analysis

Results are shown as mean ± SEM. Differences between groups were analyzed by one-way analysis of variance, and correction for multiple comparisons was made by using Tukey’s multiple comparison test. Comparison between the two groups was made by using one-sided Student’s *t*-test.

## Results

### Expression and translocation of α3NaK in human CCCs

The correlation between α3NaK mRNA expression and overall survival of gastric cancer patients was evaluated using Kaplan-Meier Plotter (www.kmplot.com) (Fig. 1A). A high level of α3NaK mRNA was correlated with poor overall survival in all patients with gastric cancer (Fig. 1A), suggesting that the aberrant expression of α3NaK in gastric cancer is closely associated with tumor malignancy and poor prognosis.

An increase in CCC numbers is correlated with a higher likelihood of metastasis and poorer prognosis in gastric cancer [13, 14]. To examine the expression of α3NaK in human CCCs, peripheral venous blood samples were collected from patients with gastric cancer. The CCCs were isolated and enriched using the RosetteSep™ Human CD45 Depletion Cocktail and Lymphoprep (Fig. 1B). The CD45 antibody used was confirmed to react with the human CD45 recombinant protein (Fig. S1A). The CD45 (-) cells expressing EpCAM were used for following experiments of CCCs (Fig. 1D). α3NaK was localized in the cytoplasm of primary gastric cancer cells (Fig. 1C), whereas in CCCs, α3NaK was colocalized with flotillin-2, a marker for PM (Fig. 1D, E). Notably, when CD45 (-) cells were attached to the poly-L-lysine-coated glass-based dish for 1 h before fixation, α3NaK was mainly localized in cytoplasm of the attached CCCs (Fig. 1D, E). α3NaK was also found in the PM of single floating cancer cells isolated by enzymatic digestion of primary gastric cancer tissues (Fig. 1F, G). When the floating cells were re-attached to the culture dish, α3NaK was returned to the cytoplasm (Fig. 1F, G). These results suggest that detachment-induced PM-translocation of α3NaK occurs in CCCs from gastric cancer patients.

### Digoxin inhibits the PM-translocation of α3NaK and promotes anoikis upon cancer cell detachment

**Fig. 2:**
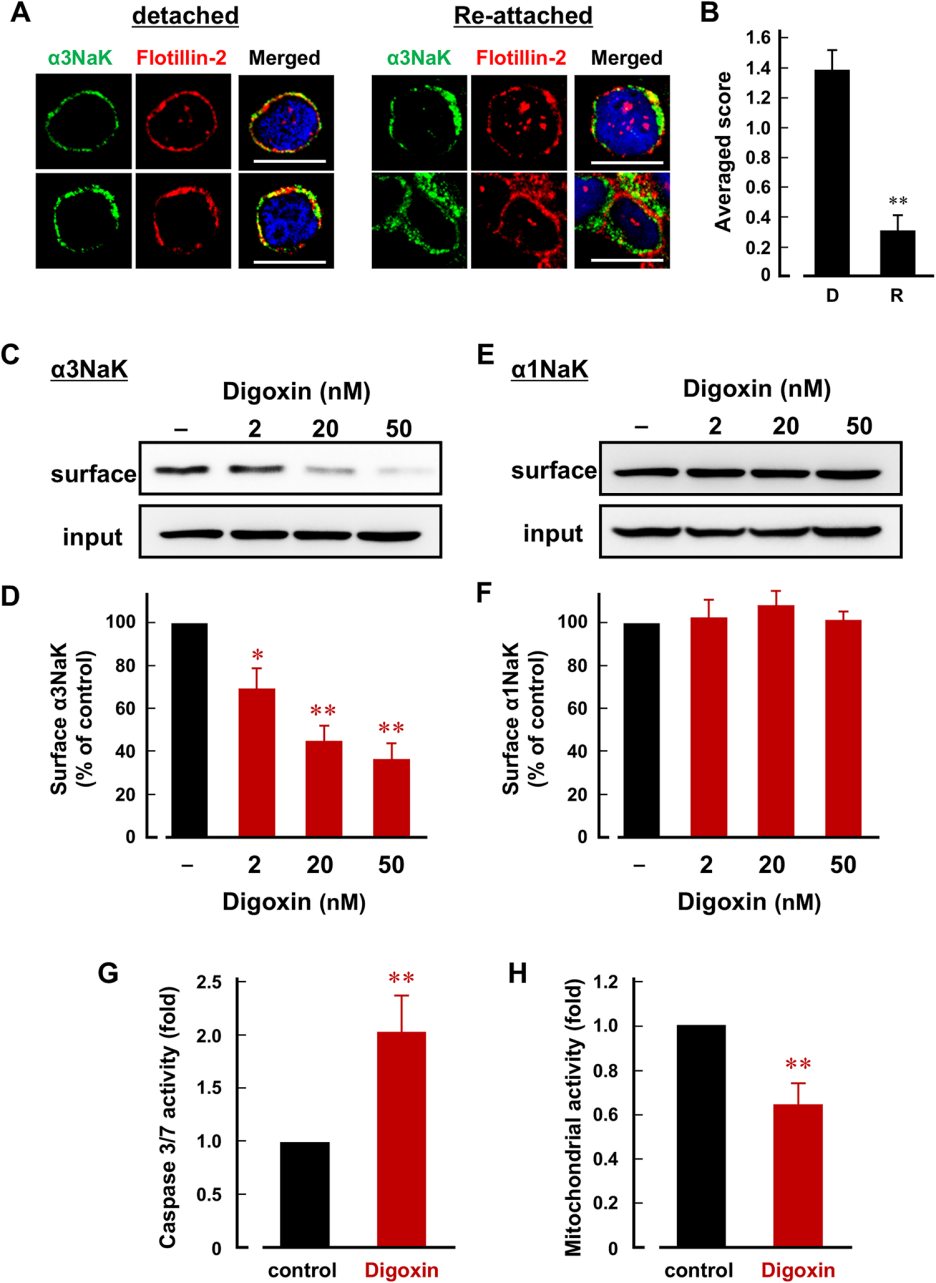
Digoxin inhibits the translocation of α3NaK from cytoplasm to the PM, and promotes anoikis in MKN45 cells. **A** Immunocytochemistry was performed using antibodies for α3NaK (green) and flotillin-2 (red) in detached and re-attached MKN45 cells. Cells were detached by the treatment with the solution containing 0.25% trypsin and 10 mM EDTA. Scale bars, 10 μm. **B** The distribution of α3NaK in detached (D) and re-attached (R) MKN45 cells were scored as 0 (predominantly at cytoplasm), 1 (both at cytoplasm and PM), and 2 (predominantly at PM) in (**A**). **C**–**F** Cell surface biotinylation was performed in detached MKN45 cells. Effects of digoxin (2, 20, and 50 nM) on the surface expression level of α3NaK in detached MKN45 cells. (-) indicates cells not treated with digoxin. Typical images of Western blots using antibodies for α3NaK (110 kDa; **C**) and α1NaK (100 kDa; **E**) in the total lysates (input) and biotinylation samples (surface) were shown. Quantification of the surface expression level of α3NaK in (**C**) and (**E**) (**D**, **F**). *n* = 3–4. *p < 0.05; **p < 0.01. **G** The effect of digoxin (20 nM) on caspase 3/7 activity of detached (floating) MKN45 cells. The activity in the digoxin-treated cells was compared with untreated cells (control) (*n* = 6). **p < 0.01. **H** Cell viability was assessed by measuring mitochondrial activity. The effect of digoxin (20 nM) on cell viability was examined in detached (floating) MKN45 cells. The activity in the digoxin-treated cells was compared with untreated cells (control) (*n* = 6). **p < 0.01.

Here, human gastric cancer MKN45 cells were used to investigate the effects of digoxin, a cardiac glycoside, on the anoikis of the cells. In attached MKN45 cells, α3NaK was localized in the cytoplasm, whereas upon cell detachment, α3NaK was translocated from cytoplasm to the PM (Fig. 2A, B). When the detached cells were re-attached to the culture dish, α3NaK returned to the cytoplasm (Fig. 2A, B).

Next, the effect of digoxin on detachment-induced PM-translocation of α3NaK was examined by surface biotinylation assay. Digoxin (2, 20, and 50 nM) dose-dependently inhibited detachment-induced PM-translocation of α3NaK without affecting the total expression level of α3NaK (Fig. 2C, D). In contrast, digoxin (2, 20, and 50 nM) had no significant effect on the expression level of α1NaK in PM (Fig. 2E, F).

To examine the effect of digoxin on anoikis, MKN45 cells were pretreated with digoxin (20 nM) for 1 h, detached with EDTA, and cultured in the presence of digoxin for 48 h. Apoptosis was assessed by measuring caspase 3/7 activity and cell viability. Digoxin significantly increased caspase 3/7 activity and reduced cell viability compared with control cells (Fig. 2G, H), suggesting that digoxin promote anoikis in the detached (floating) MKN45 cells by inhibiting the PM-translocation of α3NaK.

### Inhibition of α3NaK but not α1NaK activity by nanomolar concentrations of digoxin in cancer cells and tissues

**Fig. 3:**
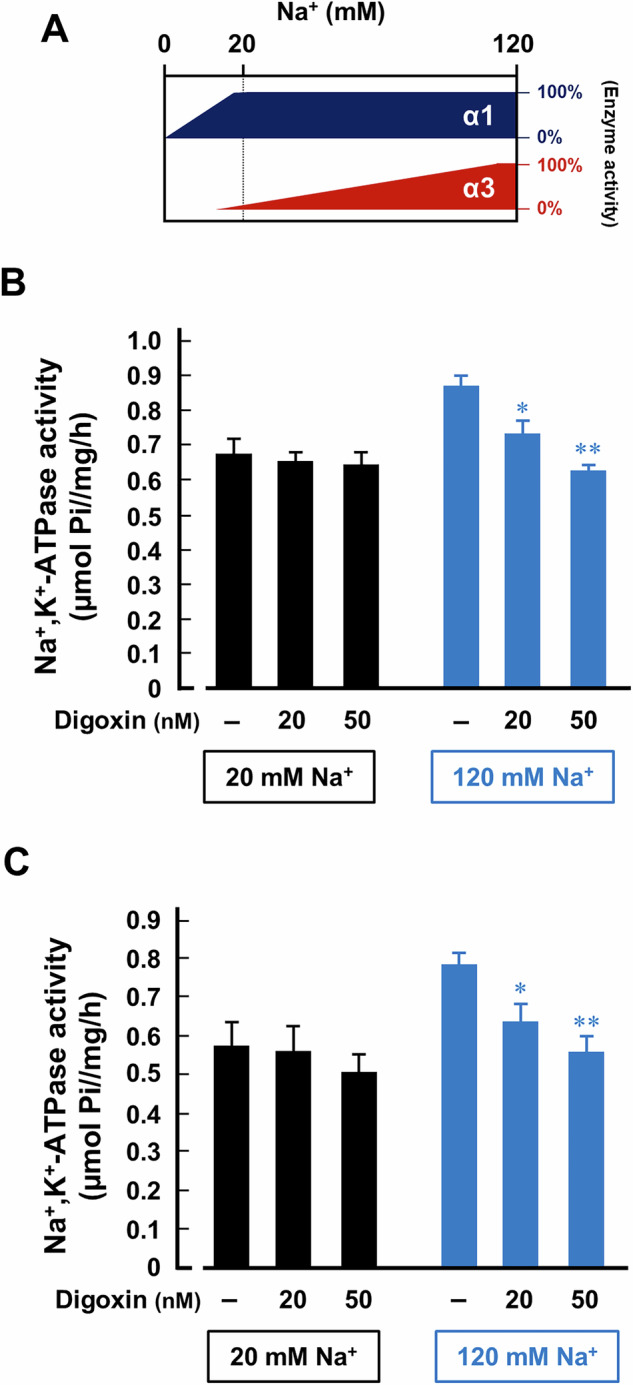
Inhibition of α3NaK activity by digoxin in MKN45 cells and gastric cancer tissues. **A** Na^+^/K^+^-ATPase activities in the samples were measured at two different Na^+^ concentrations; α1NaK predominantly contributes to total Na^+^/K^+^-ATPase activity at 20 mM of Na^+^, and both α1NaK and α3NaK contribute to total activity at 120 mM of Na^+^. Digoxin (20 and 50 nM)-sensitive Na^+^/K^+^-ATPase activities of the membrane fractions of MKN45 cells (**B**) and human gastric cancer tissues (**C**) were measured in the 20 mM-Na^+^ and 120 mM-Na^+^ solutions. (-) indicates cells not treated with digoxin. *n* = 3-4. *p < 0.05; **p < 0.01.

We examined the effects of digoxin (20 and 50 nM) on the enzyme activity of Na^+^/K^+^-ATPase under 20 and 120 mM Na^+^ conditions in the membrane fractions prepared from MKN45 cells and gastric cancer tissues. Among the different human α-isoforms of Na^+^/K^+^-ATPase, α3NaK has the lowest Na^+^ affinity. The activity of α3NaK at 20 mM Na^+^ is approximately 15% of the maximum activity obtained at 100 mM Na^+^, whereas the activity of α1NaK at 20 mM Na^+^ is approximately 100% of its maximum activity at 100 mM Na^+^ [32]. Thus, Na^+^/K^+^-ATPase activity at 20 mM Na^+^ is predominantly due to α1NaK, while at 120 mM Na^+^, it is contributed by both α1NaK and α3NaK (Fig. 3A). Digoxin (20 nM and 50 nM) had no effect on Na^+^/K^+^-ATPase activity at 20 mM Na^+^, but significantly inhibited activity at 120 mM Na^+^ in the membrane (cell-free) sample from MKN45 cells (Fig. 3B). Similarly, digoxin (20 and 50 nM) significantly inhibited Na^+^/K^+^-ATPase activity at 120 mM Na^+^ but not at 20 mM Na^+^ in the membrane sample from gastric cancer tissues (Fig. 3C). On the other hand, digoxin (20 nM) significantly increased caspase 3/7 activity and the expression of cleaved caspase 3 (17 and 19 kDa) in the detached (floating) MKN45 cells cultured in the 120 mM Na^+^-medium; however, these effects of digoxin were not observed in the 20 mM Na^+^-medium (Fig. S2). These results suggest that nanomolar concentrations of digoxin predominantly target α3NaK, rather than α1NaK, in the cancer cells. A slight difference was observed in the concentration dependency of digoxin’s effects on the surface expression of α3NaK (Fig. 2D) compared to its influence on the α3NaK-derived ATPase activity (Fig. 3B, C). This difference may be attributed to variations in experimental conditions, specifically the use of cell-based versus cell-free samples, each employing solutions with differing ion compositions.

### Expression of ECM-related components in CCCs

**Fig. 4:**
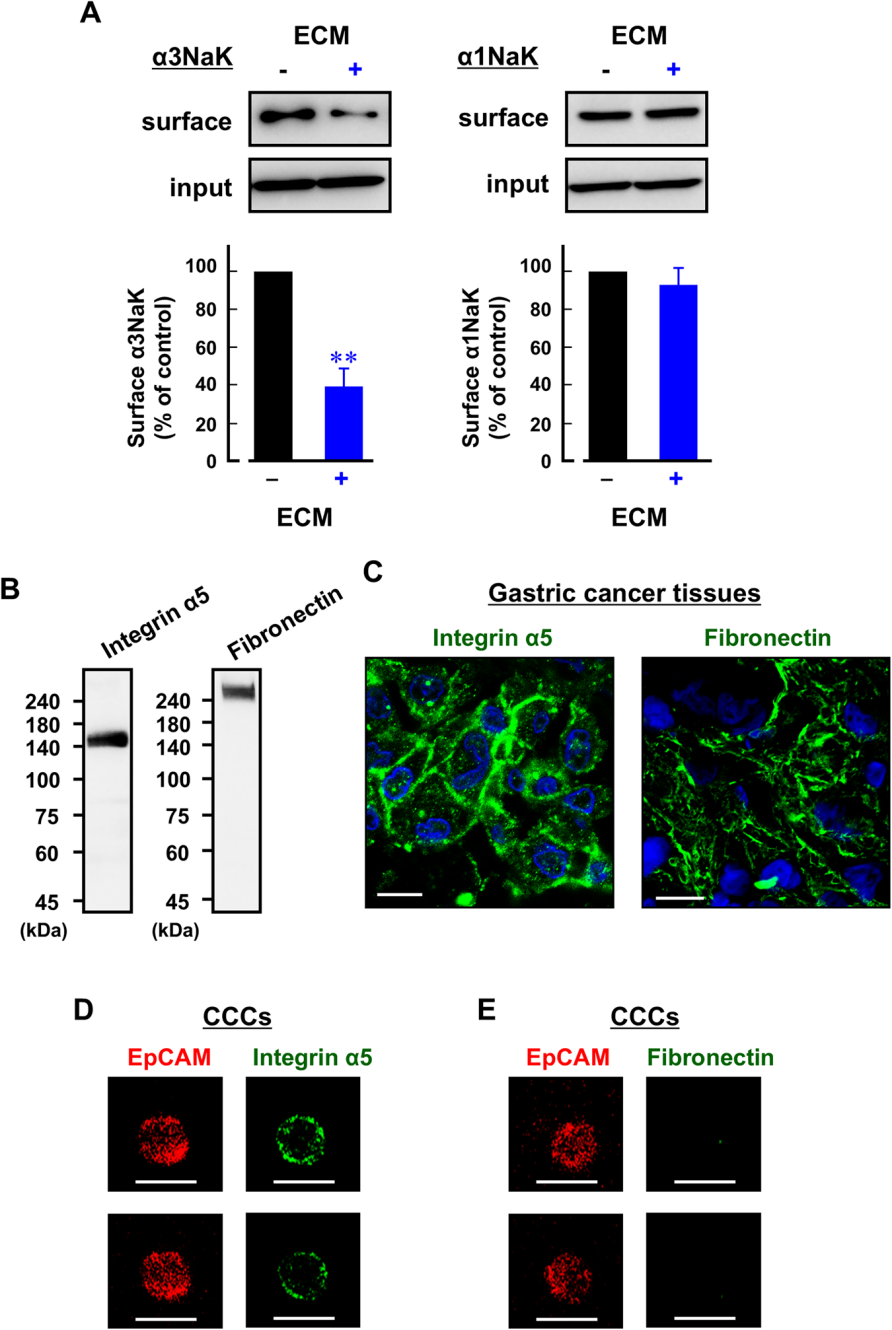
Expression of ECM-related components in CCCs. **A** Surface expression levels of α3NaK and α1NaK in detached MKN45 cells under the presence (+) and absence (−) of ECM components (fibronectin, collagen, and laminin; 15 μg/ml). Representative Western blot images using antibodies against α3NaK (110 kDa) and α1NaK (100 kDa) in the total lysates (input) and biotinylation samples (surface) were shown in the upper panels. In the lower panels, surface expression level of α3NaK and α1NaK was quantified. *n* = 4. **p < 0.01. **B** Western blotting was performed using anti-integrin α5 (140 kDa) or fibronectin (260 kDa) antibody in gastric cancer tissues. **C** Immunocytochemistry was conducted with antibodies against integrin α5 and fibronectin in gastric cancer tissues. Scale bars, 10 μm. Immunocytochemistry was performed using antibodies against EpCAM (red) and integrin α5 (**D**; green) or fibronectin (**E**; green) in CCCs from gastric patients. Scale bars, 10 μm.

Next, we examined the role of ECM in the detachment-induced α3NaK translocation. Addition of ECM components (fibronectin, collagen, and laminin; 15 μg/ml) to the extracellular solution significantly decreased the surface expression of α3NaK in detached MKN45 cells (Fig. 4A), suggesting that α3NaK translocation is regulated by cell-ECM interaction in gastric cancer cells.

Integrins, a superfamily of cell adhesion receptors, bind to the ECM and play important roles in various aspects of cancer progression, including proliferation, migration, invasion, and metastasis [33, 34]. Among these, integrin α5β1, a heterodimer comprising α5 and β1 subunits, serves as a receptor for fibronectin and is implicated in cancer metastasis, including gastric cancer [35–37]. In gastric cancer tissues from patients, integrin α5 and fibronectin were both detected (Fig. 4B, C). Interestingly, while integrin α5 was present in CCCs from the patients, fibronectin was not (Fig. 4D, E). These results suggest that CCCs from gastric cancer patients retain integrin α5 expression, which allows binding to fibronectin and promotes CCC aggregation [38, 39].

### Digoxin suppresses liver metastasis by reducing CCCs

**Fig. 5:**
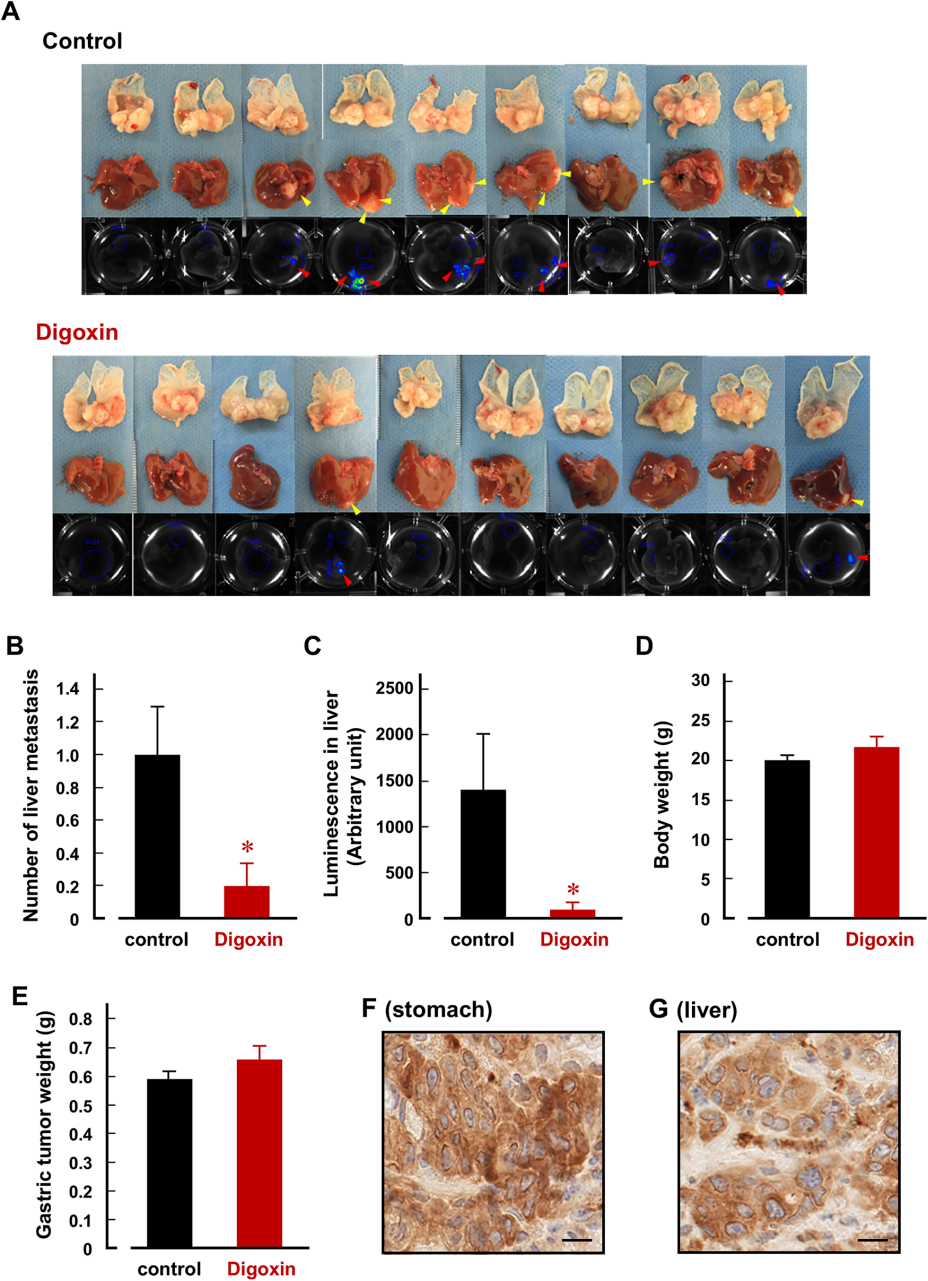
Digoxin inhibits liver metastasis in the MKN45 orthotopic xenograft model. **A** The series of images showed the growth of transplanted gastric cancers in the stomach (top) and their metastasis to the liver (middle) in each orthotopic xenograft mouse treated with or without digoxin (2 mg/kg/day). Ex vivo bioluminescence imaging of the liver (bottom) further identifies liver metastases. Arrowheads indicate metastatic gastric cancers in the liver. **B** The numbers of liver metastasis were compared between control (*n* = 9) and digoxin-treated (*n* = 10) mice. *p < 0.05. **C** Bioluminescence intensities were quantified ex vivo in the liver of control (*n* = 9) and digoxin-treated (*n* = 10) mice. *p < 0.05. **D** Body weights were compared between control (*n* = 9) and digoxin-treated (*n* = 10) mice. **E** Tumor weights isolated from stomachs were compared between control (*n* = 9) and digoxin-treated (*n* = 10) mice. Immu*n*ohistochemistry using anti-α3NaK antibody was performed in primary gastric cancer tissues in the stomach (**F**) and metastatic cancer tissues in the liver (**G**). Scale bars, 10 μm.

Here, we established a xenograft model of liver metastasis by injecting MKN45 cells, which stably express luciferase, into the stomachs of nude mice (BALB/c-nu/nu). Six weeks after injection, the mice were intraperitoneally injected with digoxin (2 mg/kg/day) for 2 weeks. Based on a previous report [40], the plasma concentrations of digoxin in mice are estimated to be approximately the therapeutic range for humans (0.5-2 ng/ml). Interestingly, digoxin significantly decreased the number of liver metastasis in treated mice compared with non-treated control mice (Fig. 5A, B). Luminescence in the livers of digoxin-treated mice was also significantly lower than control mice (Fig. 5A, C). On the other hand, the body weight and wet weight of primary gastric cancer tissues were comparable between the control and digoxin-treated mice (Fig. 5D, E). In the isolated stomachs and livers, α3NaK was localized in the cytoplasm of the cancer cells (Fig. 5F, G).

**Fig. 6:**
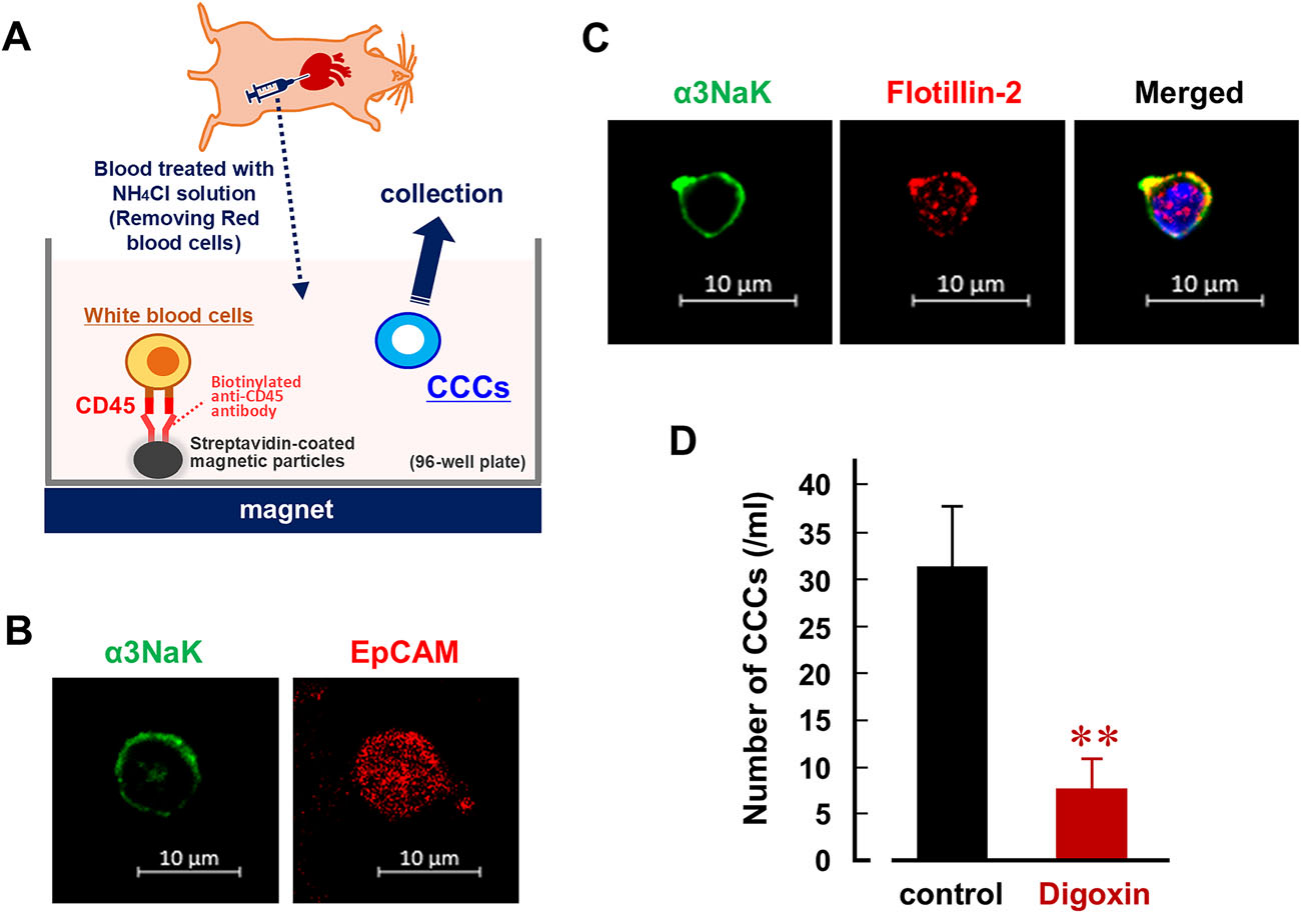
Digoxin decreases the number of CCCs in the MKN45 orthotopic xenograft model. **A** A schematic overview of the experimental procedure for isolation of CCCs from mice. Immunocytochemistry was performed using antibodies for α3NaK (green), EpCAM (red; **B**), and flotillin-2 (red; **C**) in CCCs. Scale bars, 10 μm. **D** The number of CCCs was compared between control (*n* = 9) and digoxin-treated (*n* = 10) mice. **p < 0.01.

Next, CCCs were isolated from the whole blood of mice using a CD45-based negative immunomagnetic separation procedure (Fig. 6A). The CD45 antibody used was confirmed to react with the mouse CD45 recombinant protein (Fig. S1B). EpCAM-expressing CD45 (-) cells were used for the experiments (Fig. 6B). Similar to human CCCs isolated from gastric cancer patients (Fig. 1D), α3NaK was predominantly localized in the PM of CCCs (Fig. 6C). Notably, the administration of digoxin significantly reduced the number of CCCs (control mice: 31.3 ± 6.4/ml vs. digoxin-treated mice: 7.6 ± 3.2/ml) (Fig. 6D).

**Fig. 7:**
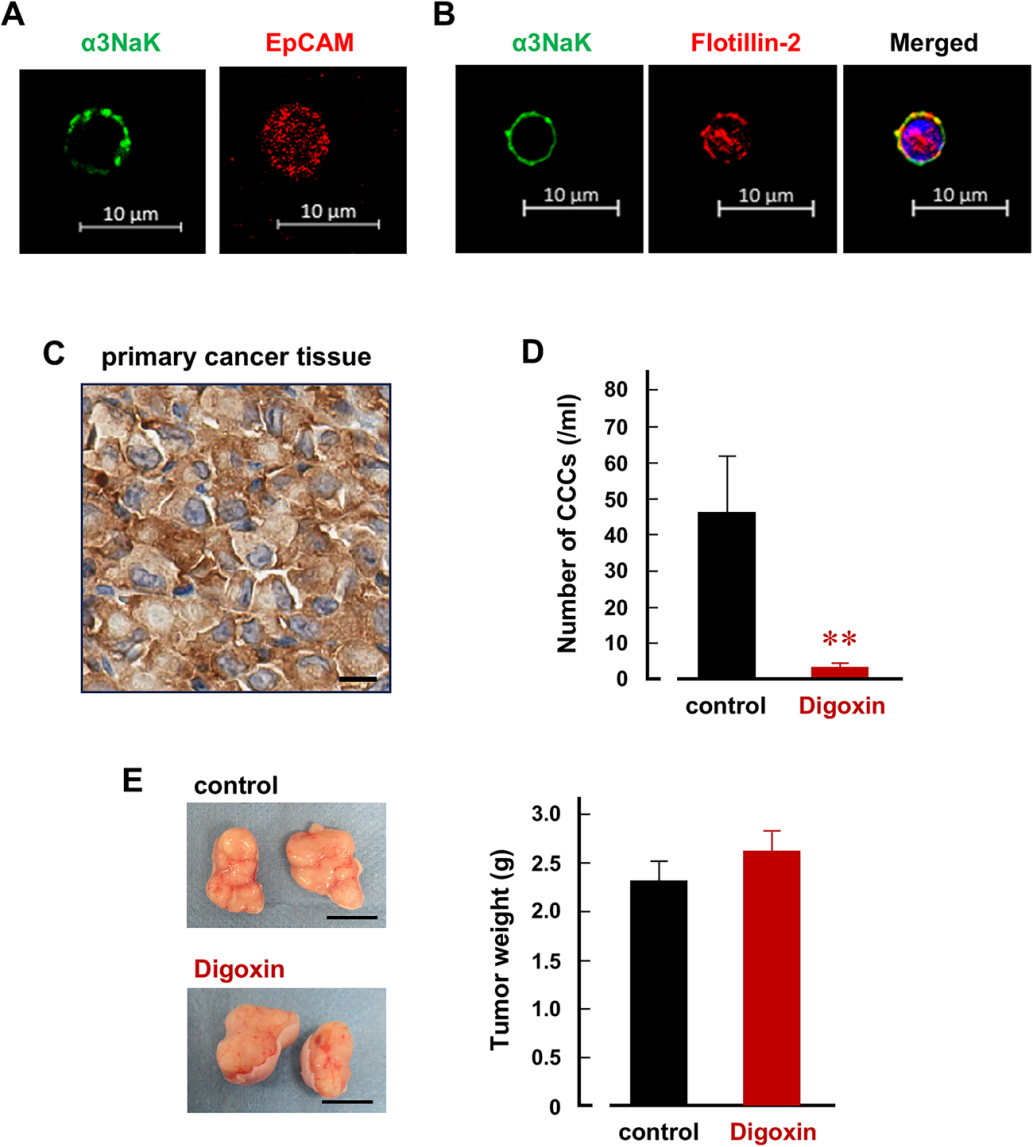
Digoxin decreases the number of CCCs in the MKN45 subcutaneous xenograft model. Immunocytochemistry was performed using antibodies for α3NaK (green), EpCAM (red; **A**), and flotillin-2 (red; **B**) in CCCs. **C** Immunohistochemistry using anti-α3NaK antibody was performed in subcutaneous cancer tissue derived from plantation of MKN45 cells. Scale bars, 10 μm. **D** The number of CCCs was compared between control (*n* = 7) and digoxin-treated (*n* = 6) mice. **p < 0.01. **E** Typical images of subcutaneous cancer tissues derived from plantation of MKN45 cells were shown (left). Tumor weights of the subcutaneous cancer tissues were compared between control (*n* = 7) and digoxin-treated (*n* = 6) mice (right).

Furthermore, we investigated the efficacy of digoxin in inhibiting CCCs using a mouse subcutaneous xenograft model. EpCAM-expressing CD45 (-) cells were used for the CCC experiments (Fig. 7A). α3NaK was localized in the PM of CCCs (Fig. 7B), while in primary cancer tissues, it was expressed in the cytoplasm of cancer cells (Fig. 7C). The administration of digoxin (2 mg/kg/day) for 1 week significantly decreased the number of CCCs (control mice: 46.2 ± 15.1/ ml vs digoxin-treated mice: 3.2 ± 1.0/ ml) (Fig. 7D). Conversely, no significant differences were observed in the wet weights of subcutaneous tumors between the control and digoxin-treated mice (Fig. 7E).

## Discussion

After cancer cells detach from their primary cancer tissues in the process of distant metastasis, CCCs must overcome anoikis, shearing forces, and immune surveillance during circulation in the bloodstream [41]. Anoikis is induced by losing anchorage from the ECM, but detached cancer cells have the anoikis-resistance mechanism to compensate for initial step of cancer metastasis [17]. Recently, we found that the translocation of α3NaK from cytoplasm to the PM is required for survival of the detached (floating) cancer cell lines in vitro [22], and that the activation of AMP-activated protein kinase (AMPK), which is an essential regulator of anoikis resistance [40, 41], occurs downstream of the PM translocation of α3NaK [22]. In the present study, we showed for the first time that α3NaK is localized in the PM of CCCs isolated from patients with gastric cancer and MKN45 xenograft mouse models in vivo. Notably, α3NaK was moved from the PM to the cytoplasm, when the CCCs were attached to culture dishes (Fig. 1). Here, we employed EpCAM as one of CCC markers to isolate CCCs from the samples, and then found expression of α3NaK in almost all EpCAM-positive cells.

**Fig. 8:**
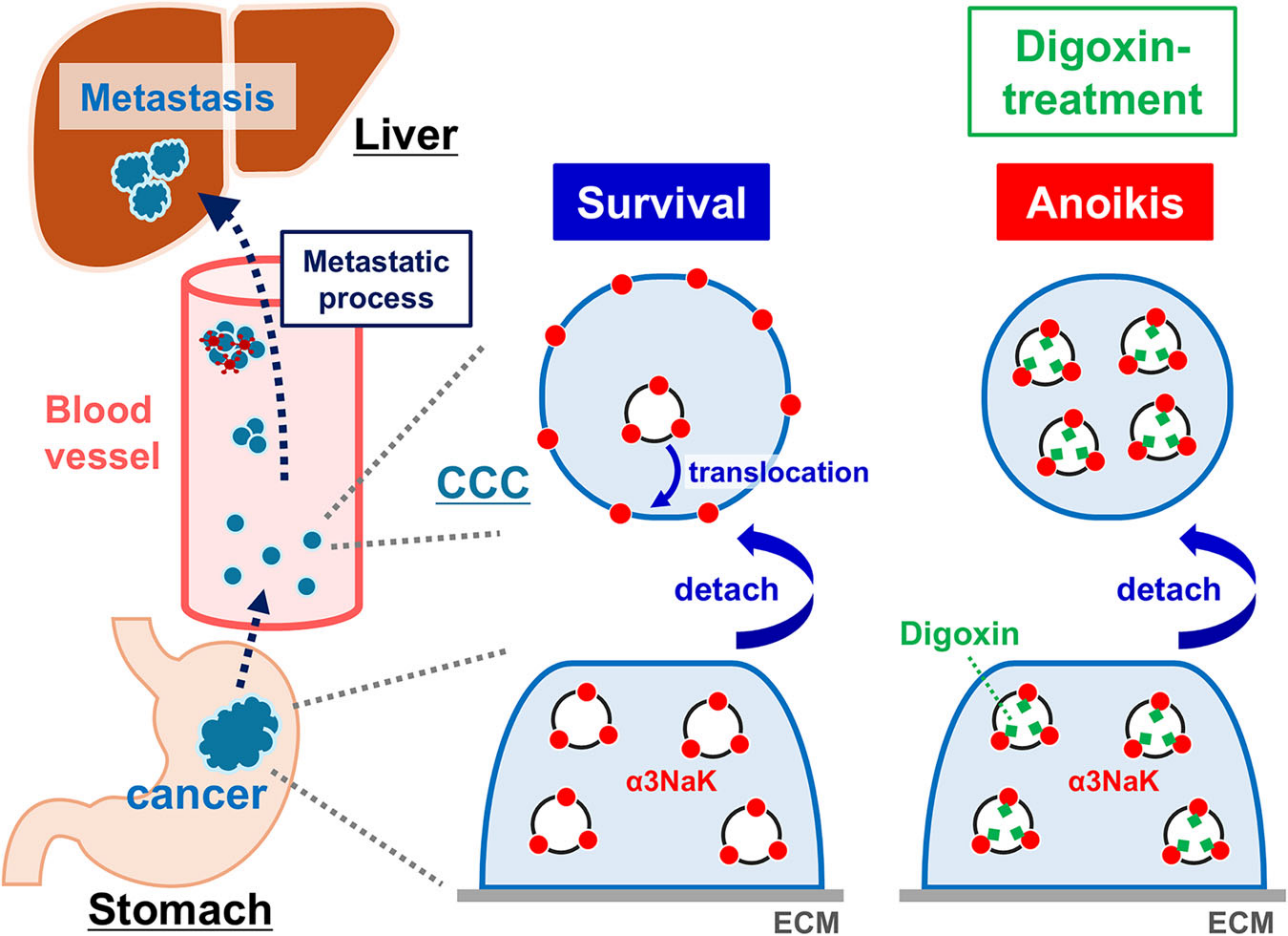
Scheme of the effect of digoxin on the anoikis resistance of CCCs. Digoxin inhibits the translocation of α3NaK from cytoplasm to the PM upon cancer cell detachment, and promote anoikis in the CCCs, suppressing liver metastasis of gastric cancer.

Digoxin (20 and 50 nM) selectively inhibited α3NaK activity in human gastric cancer tissues and MKN45 cells, without affecting α1NaK (Fig. 3), suggesting that affinity of digoxin for intracellular α3NaK is higher than α1NaK in the PM. In fact, ouabain (200 nM) significantly inhibited the enzyme activity of α3NaK but not α1NaK in human liver cancer HepG2 cells [29]. Digoxin also inhibited the α3NaK translocation from cytoplasm to the PM and promoted apoptosis in the detached (floating) MKN45 cells (Fig. 2). In the MKN45 xenograft mouse models in vivo, intraperitoneal administration of digoxin (in therapeutic range for humans) markedly decreased the number of CCCs and inhibited liver metastasis (Figs. 5 and 6). We suggest that α3NaK localized in the PM is crucial for survival of CCCs, and that the digoxin-induced inhibition of the α3NaK translocation (from the cytoplasm to the PM) suppresses the production of CCCs from primary cancer tissues and the survival of CCCs in circulation (Fig. 8).

On the other hand, nanomolar concentrations of digoxin have been shown to modulate the nuclear receptor (RORγ)-dependent transcription and several cellular signaling pathways, including HIF-1, Src, NF-κB, and EGFR-STAT3, thereby contributing to its anti-cancer activities [42, 43]. Low concentrations (<1 nM) of cardiac glycosides have been reported to stimulate the proliferation of vascular smooth muscle cells, umbilical vein endothelial cells, and kidney cells, by activating Na^+^/K^+^-ATPase and inducing signal transduction pathways, such as Src and PI3K-Akt [42, 44]. Further research is necessary to determine whether the signaling pathways activated by digoxin contribute to the survival of CCCs.

In the present study, we showed that in the initial step of metastasis, the loss of cell-ECM interaction induces the PM-translocation of α3NaK for anoikis resistance (Fig. 4A). Conversely, fibronectin, an ECM component, has been reported to be involved in CCC aggregation and survival, thereby promoting cancer metastasis [38, 39, 45]. Among integrins, integrin α5β1 serves as a fibronectin receptor in various cancers, including gastric cancer [35–37]. Here, we found that CCCs from gastric cancer patients retain the expression of integrin α5 (Fig. 4D), while fibronectin was not detected in CCCs (Fig. 4E). Collectively, gastric cancer cells that detach from the primary tumor lose their ECM components, leading to the migration of intracellular α3NaK to the PM as a mechanism to evade anoikis. In the later stages of metastasis, fibronectin may subsequently bind to CCCs, potentially facilitating CCC clustering [38, 39].

So far, several CCC-targeting strategies for cancer treatment have been proposed; 1) inhibition of cancer cell intravasation using EpB2, PLK1 inhibitor, and anti-integrin antibody, 2) dissociation of CCC clusters or prevention of their formation using HPSE inhibitors, Na^+^/K^+^-ATPase inhibitors, platelet receptor inhibitors, and urokinase, 3) interference with metabolism, homeostasis, and immune cell-mediated survival, 4) immune checkpoint inhibition using anti-CD47, anti-PDL1/PD1, and anti-CTLA4 antibodies, 5) use of engineered CCCs as therapeutic vehicles, and 6) CCC-based chronotherapy [46]. Among them, Gkountela et al. reported that cardiac glycosides (Na^+^/K^+^-ATPase inhibitors) such as ouabain and digitoxin dissociate CCC clusters into single cells, thereby suppressing metastasis in human breast CCC-derived cell lines (BR16 cells) [47]. Furthermore, digoxin is now under investigation in a first-in-human, proof-of-concept, therapeutic exploratory phase I trial to determine its potential to disrupt CCC clusters in patients with advanced or metastatic breast cancer (NCT03928210) [34, 46], and the trial has shown that it reduces mean CCC cluster size [48]. Thus, digoxin may play dual roles in the mechanism of cancer metastasis: one is dissociation of CCC clusters in the downstream mechanism, and another is induction of CCC anoikis in the upstream mechanism as we found in this study.

Regarding the binding site of digoxin in Na^+^/K^+^-ATPase, Ren et al. [42, 43] suggest that digoxin binds to human α1NaK with its hydroxy substituent at the C-12 position pointing to a small pocket on the side of the binding cavity, known as the Asn130 pocket, located between the αM2 and αM4 helices, with the αM1 helix. This pocket consists of several hydrophobic residues, including Ala115, Ile323, Ile326, Leu133, and Val330, with the polar residue, Asn130, located in the deeper region. Recently, cryo-EM analysis revealed the transport cycle and gating mechanism of human α3NaK [49], although the digoxin-binding structure of α3NaK has not been determined. Given that the hydrophobic residues forming the Asn130 pocket in α1NaK are conserved in α3NaK, it is suggested that digoxin binds to the pocket in α3NaK similarly to its interaction with α1NaK. However, our present study shows that translocation of α3NaK from cytoplasm to the PM is inhibited by nanomolar concentrations of digoxin, which has no effect on ubiquitous α1NaK. In addition, intracellular α3NaK of cancer cells (Fig. 3) has a higher affinity for digoxin than normal α3NaK localized in the PM of neural cells [50]. The mechanism behind why digoxin has a high affinity for α3NaK in cancer cells remains unclear at present. One possible explanation could be related to the distinct cellular environment surrounding α3NaK in cancer cells.

In conclusion, our findings suggest that targeting α3NaK may be able to specifically induce anoikis in CCCs, potentially providing an effective therapy.

## Supplementary information

Supplemental Figures

Full and uncropped western blots

Data set

## Data Availability

All data generated or analyzed during this study are included in this published article.
