# Supplementary material for: Digoxin promotes anoikis of circulating cancer cells by targeting Na+/K+-ATPase α3-isoform
Source: Cell Death Dis. 2025 May 11;16(1):373. doi: 10.1038/s41419-025-07703-z (PMC12066707; doi:10.1038/s41419-025-07703-z)
Supplement: Supplementary file 1 — Supplemental Figures [file 41419_2025_7703_MOESM1_ESM.pdf]

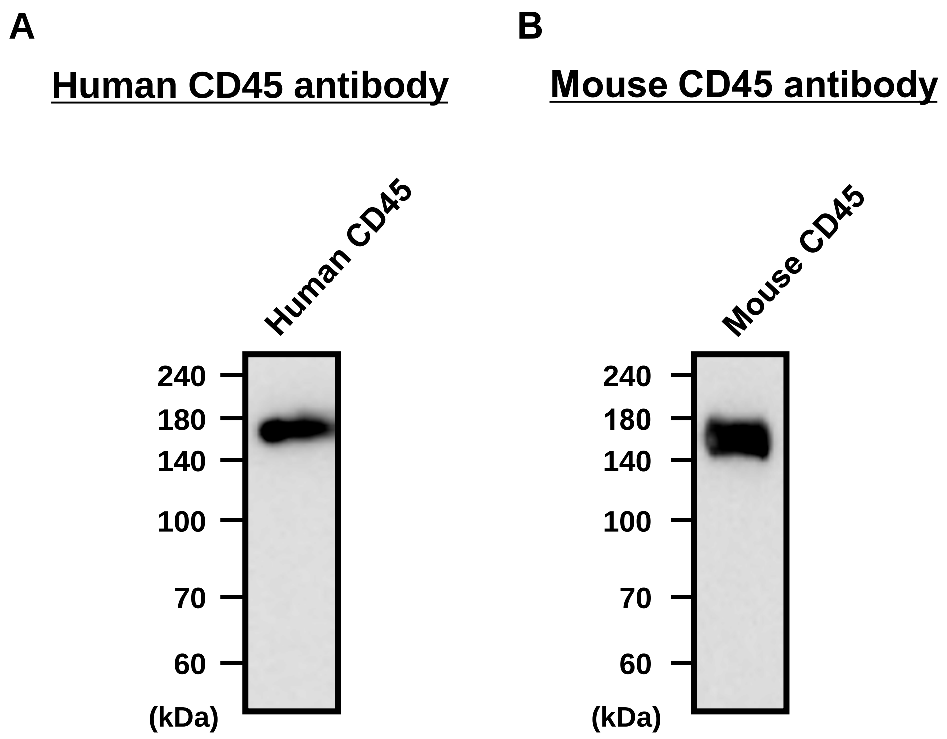

Fig. S1: Confirmation of the reactivity of human and mouse CD45 antibodies. Western blotting was performed using anti-human CD45 (A) and anti-mouse CD45 (B) antibodies on human (~170 kDa) and mouse CD45 (~160 kDa) proteins derived from the mouse myeloma cell line (NS0). Typical images were shown.

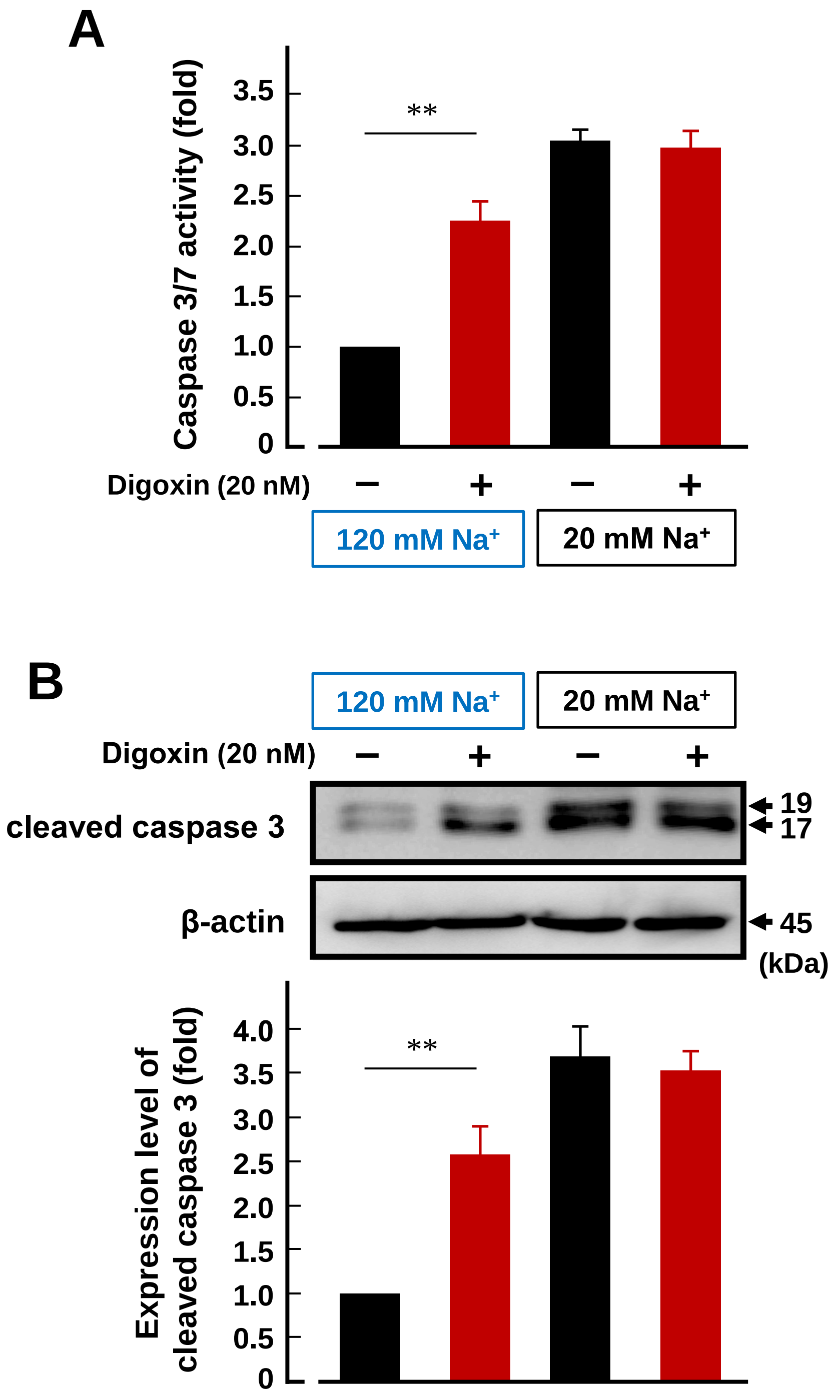

Fig. S2: Effects of digoxin on apoptosis in detached MKN45 cells.

A Effect of digoxin (20 nM) on caspase 3/7 activity of detached MKN45 cells cultured in medium containing 120 mM Na^+ or 20 mM Na^+. The activity in the digoxin-treated cells was compared with untreated cells (control). $n = 6$. **, $p < 0.01$. **B** Effects of digoxin (20 nM) on the expression level of cleaved caspase 3 in detached MKN45 cells cultured in medium containing 120 mM Na^+ or 20 mM Na^+. Typical images of Western blots using antibodies against cleaved caspase 3 (17 and 19 kDa) and β-actin (45 kDa) in the cell lysates were shown. In the lower panel, the expression levels of cleaved caspase-3 were quantified and normalized to those of β-actin. $n = 5$. **$p < 0.01$.
