# Supplementary figures and images for: Digoxin promotes anoikis of circulating cancer cells by targeting Na+/K+-ATPase α3-isoform
Source: Cell Death Dis. 2025 May 11;16(1):373. doi: 10.1038/s41419-025-07703-z (PMC12066707; doi:10.1038/s41419-025-07703-z)

Fig. 2C

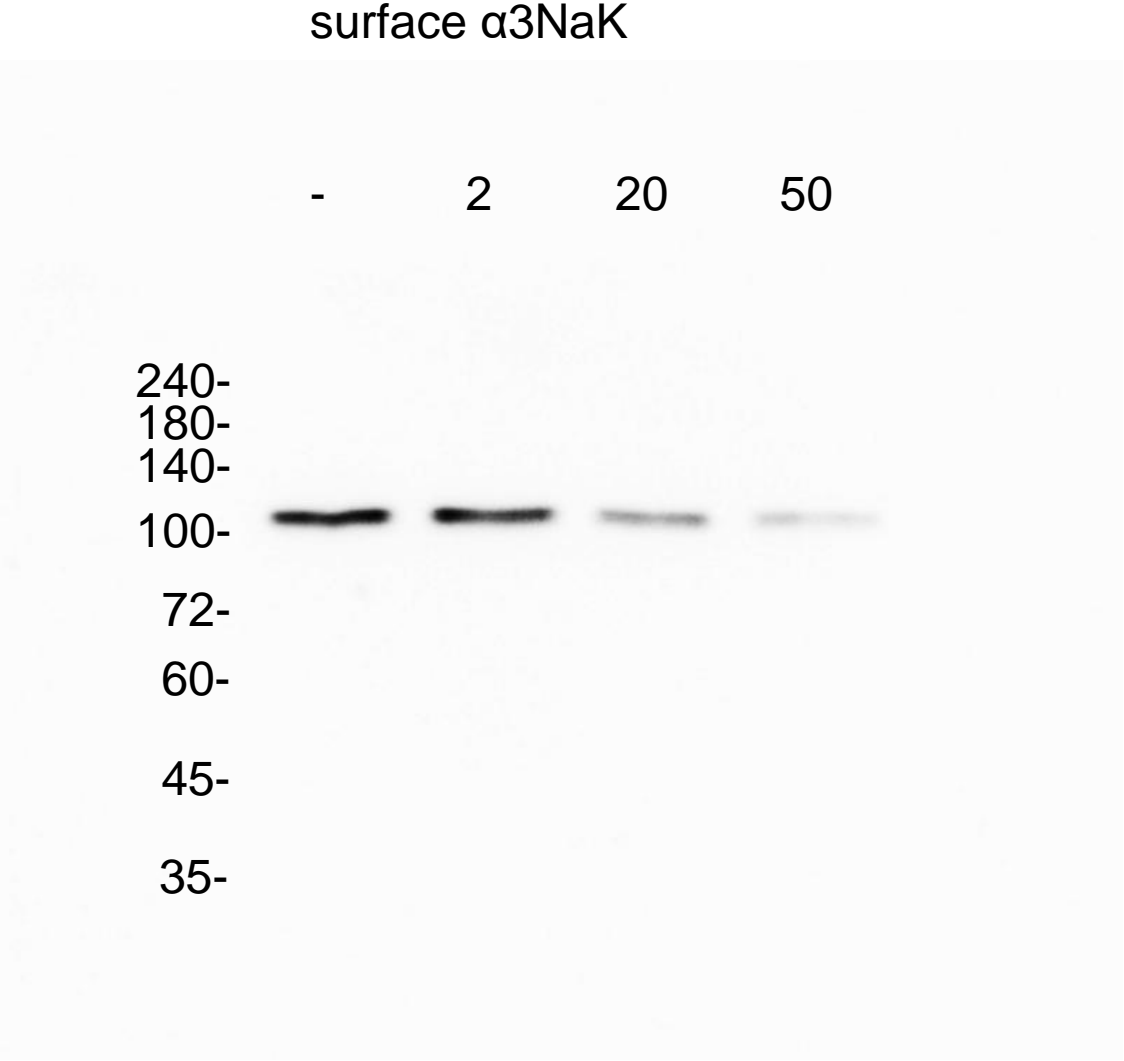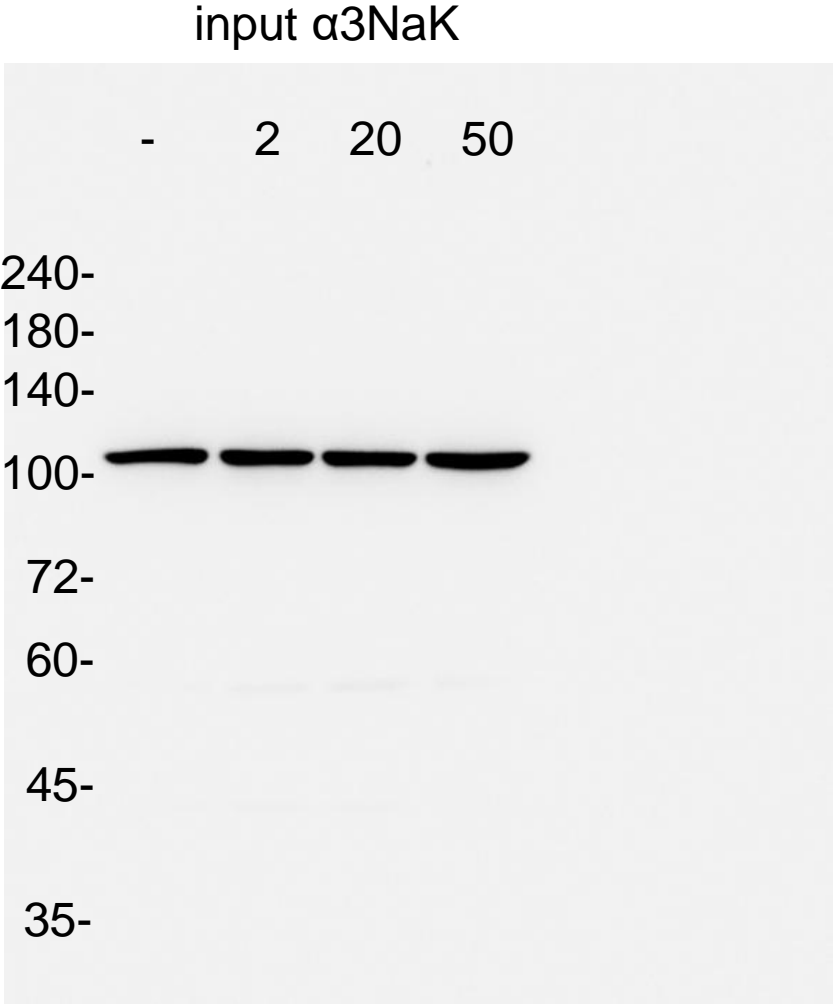

Fig. 2E

surface  $\alpha 1\text{NaK}$

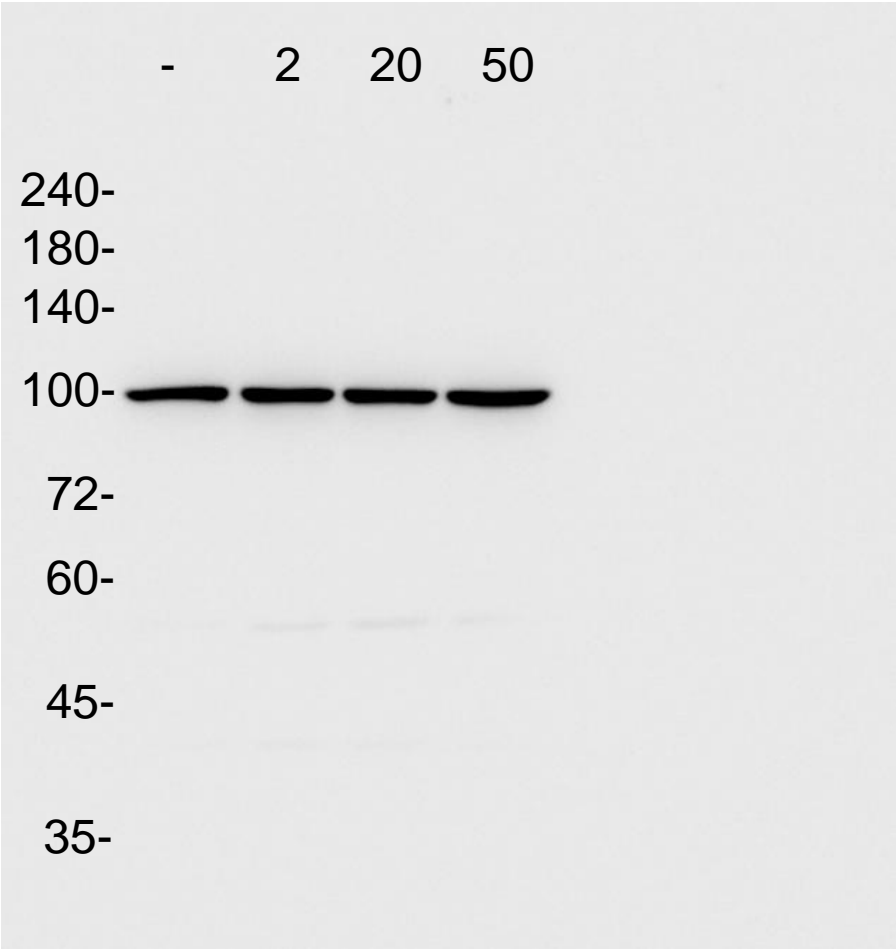

input  $\alpha 1\text{NaK}$

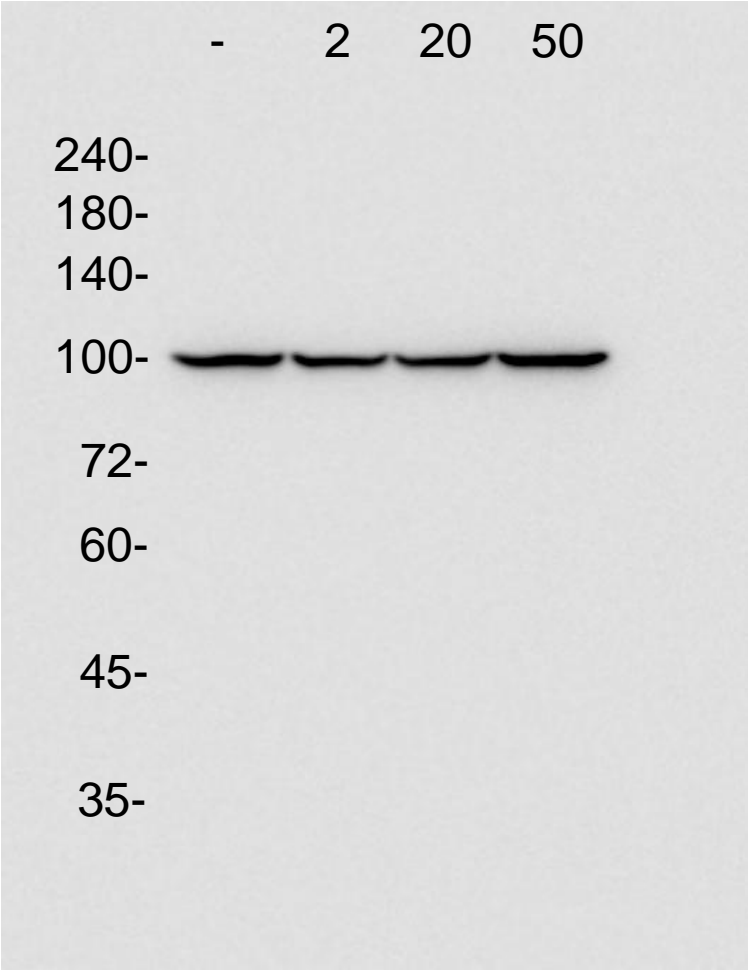

Fig. 4 A

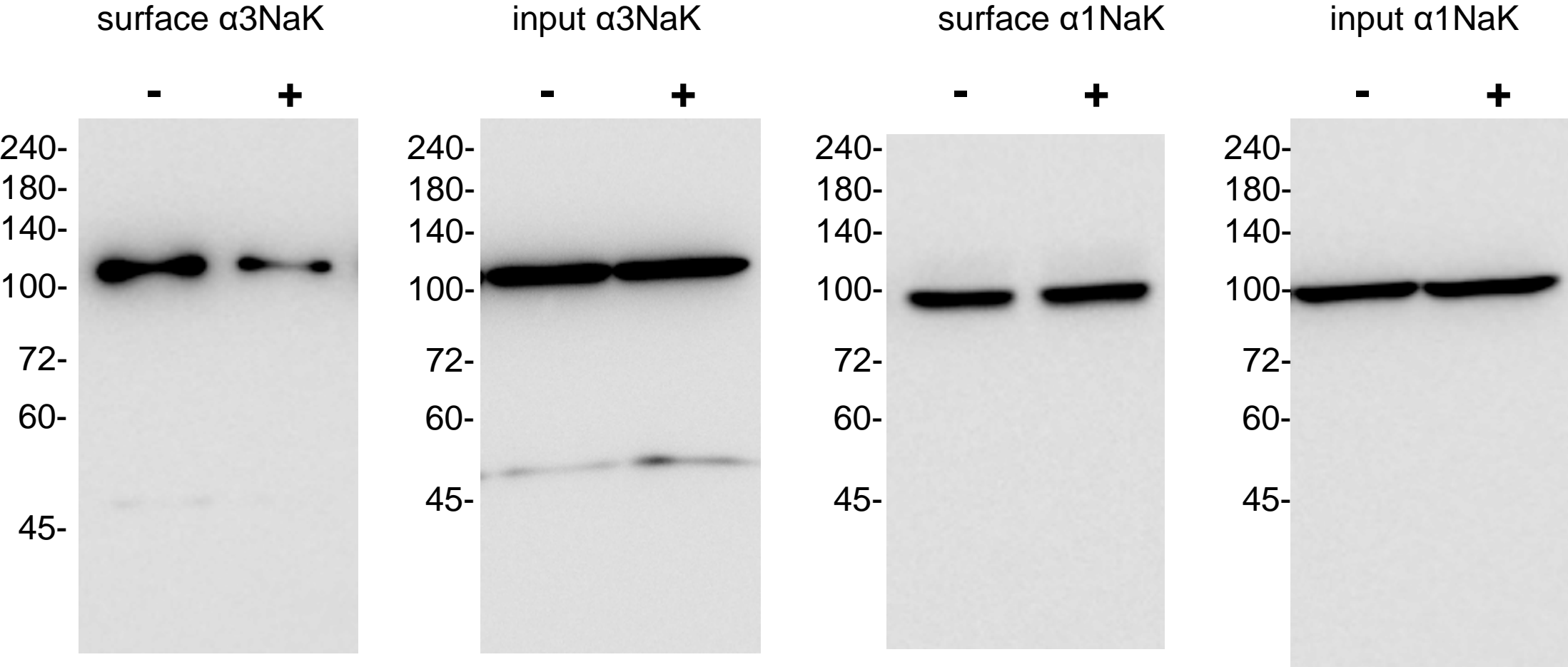

Fig. S2B

cleaved caspase 3

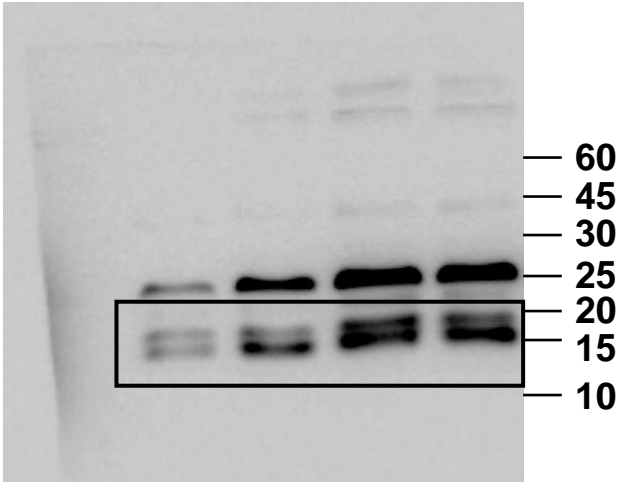

$\beta$ -actin

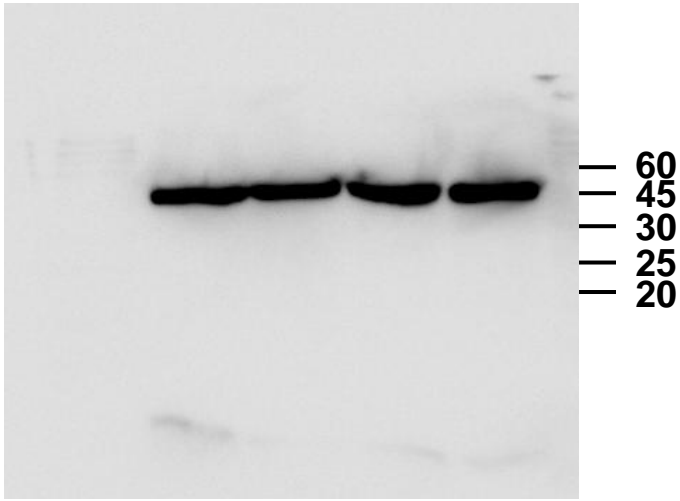

Supplement: Supplementary file 2 — Full and uncropped western blots [file 41419_2025_7703_MOESM2_ESM.pdf]
