# Supplementary material for: Digoxin promotes anoikis of circulating cancer cells by targeting Na+/K+-ATPase α3-isoform
Source: Cell Death Dis. 2025 May 11;16(1):373. doi: 10.1038/s41419-025-07703-z (PMC12066707; doi:10.1038/s41419-025-07703-z)
Supplement: Supplementary file 3 — Data set [file 41419_2025_7703_MOESM3_ESM.pdf]

PM score
human CTC

#	Detached	#	Re-attached
1	2	1	0
2	0	2	1
3	2	3	0
4	2	4	2
5	1	5	0
6	2	6	1
7	1	7	1
8	2	8	1
9	1	9	0
10	2	10	0
11	1	11	2
12	1	12	0
13	2	13	1
14	1	14	0
15	1	15	1
16	0	16	1
17	2	17	1
18	0	18	0
		19	1
		20	0
		21	1
		22	1
		23	0
		24	1
		25	1
		26	1
		27	2
		28	0
		29	0

	average	SD	SE
Detach	1.2777778	0.7307192	0.1722322
Re-attach	0.6896552	0.6487892	0.1204771

PM trance score
Human GC single cell

#	Detached	#	Re-attached
1	1	1	0
2	1	2	0
3	0	3	0
4	1	4	0
5	1	5	0
6	0	6	0
7	0	7	1
8	0	8	2
9	1	9	0
10	1	10	0
11	1	11	0
12	1	12	0
13	1	13	0
14	1	14	0
15	0	15	1
16	1	16	0
17	0	17	1
18	0	18	0
19	1	19	0
20	0	20	0
21	2	21	0
22	0	22	0
23	1	23	0
24	1	24	1
25	0	25	0
26	1	26	0
27	0	27	2
28	1	28	1
29	0	29	0
30	1	30	0
31	1	31	1
32	1	32	0
33	0	33	0
34	1	34	1
35	1	35	0
36	1	36	0
37	0	37	0
38	1	38	0
39	1	39	0
40	0	40	0
41	0	41	0
42	2	42	0
43	2	43	0
44	0	44	0
45	1	45	1
46	1	46	0
47	1	47	0
48	0	48	0
49	0	49	1
50	1	50	0
51	2	51	1
52	0	52	0
53	2	53	0
54	1	54	0
55	1	55	0
56	1	56	0
57	1	57	1
58	0	58	1
59	0	59	0
60	0	60	0
61	1	61	0
62	2	62	0
63	0	63	0
64	0	64	2
65	2	65	0
		66	0
		67	0
		68	0
		69	0
		70	0
		71	2
		72	0
		73	0
		74	0
		75	0
		76	0
		77	1
		78	0
		79	2
		80	0
		81	0
		82	2
		83	1

	average	SD	SE
Detach	0.7230769	0.3343907	0.0414761
Re-attach	0.313253	0.5997532	0.0658315

detach		scores	
9	2	2	1
10	2		
13	2	1	0
14	2		
15	1		
16	1.5		
18	2		
20	2		
22	1		
23	2	1	
25	1		
26	1		
27	1		
28	2	0	
30	1		
31	2		
32	1.5		
	AVE	SE	
	1.3913043	0.1332051	

re-atttach		scores		
36	0	0	0	1
37	0	1	0	
38	0	0		
40	0			
41	1	0		
42	0	0		
43	0			
44	1	1		
45	0	1		
46	0			
47	0			
48	1	0		
	AVE	SE		
	0.3043478	0.0981002		

	AVE	SE
detach	1.39	0.13
Reattach	0.3	0.1

a3NaK

	0	2	20	50	digoxin (nM)
1	1	0.423098	0.250198		
2	1	0.572931	0.557832		
3	1	0.716258	0.304477	0.215273	
5	1	0.948074	0.564593	0.429408	
6	1	0.826465	0.564593	0.459879	
AVE	1	0.697365	0.448339	0.368187	
SE	0	0.092354	0.070347	0.076961	

a1NaK

	0	2	20	50	digoxin (nM)
2	1	1.133704	1.136168		
3	1	0.759854	0.987094		
4	1	0.92248	1.020708	1.07426	
5	1	1.072505	0.920975	1.00251	
6	1	1.204421	1.31374	0.950211	
AVE	1	1.018593	1.075737	1.008994	
SE	0	0.079647	0.068969	0.035956	

	1	2	3	4	5	6
control	1158	910	741.5	1666	715	1041.5
digoxin	1474.5	1285	1203.5	2764	2549	2683

							AVE	SE
control	1	1	1	1	1	1	1	0
digoxin	1.273316	1.412088	1.623061	1.659064	3.565035	2.576092	2.018109	0.361076

MTT

digoxin	1	2	3	4	5	6
+	1810	1060	1790	2660	1830	2310
	650	1240	1890	2250	2410	2030
AVE	1230	1150	1840	2455	2120	2170
−	2750	3770	2120	3260	2480	2620
	2690	2900	3420	2940	2520	2410
AVE	2720	3335	2770	3100	2500	2515

							AVE	SE
-	1	1	1	1	1	1	1	0
+	0.4522059	0.3448276	0.6642599	0.7919355	0.848	0.8628231	0.6606753	0.0701807

MKN45

Na+	digoxin (nM)					AVE	SE
20.000	-	0.617	0.715	0.710		0.681	0.032
	20	0.646	0.607	0.705		0.653	0.029
	50	0.696	0.587	0.656		0.646	0.032
120.000	-	0.879	0.819	0.908	0.908	0.878	0.021
	20	0.711	0.705	0.834	0.712	0.741	0.031
	50	0.622	0.612	0.642	0.652	0.632	0.009

human gastric cancer

Na+	digoxin (nM)				AVE	SE
20.000	-	0.498	0.686	0.547	0.577	0.056
	20	0.469	0.676	0.537	0.561	0.061
	50	0.424	0.573	0.508	0.501	0.043
120.000	-	0.765	0.839	0.750	0.785	0.028
	20	0.553	0.691	0.666	0.637	0.043
	50	0.498	0.563	0.617	0.559	0.034

a3NaK

		intensity	(-BG)	fold
ECM	-	36.063	19.844	
	+	28.165	11.946	0.6019956
	background	16.219		

ECM	-	53.378	35.318	
	+	24.475	6.415	0.1816354
ECM	-	50.868	32.808	
	+	28.174	10.114	0.3082785
	background	18.06		

ECM	-	87.647	75.295	
	+	46.731	34.379	0.4565907
	background	12.352		

	1	0.456591
	1	0.601996
	1	0.181635
	1	0.308278
AVE	1	0.387125
SE	0	0.107821

a1NaK

		intensity	(-BG)	fold
ECM	-	59.494	40.713	
	+	62.663	43.882	1.0778375
	background	18.781		

ECM	-	71.857	55.206	
	+	58.469	41.818	0.7574901
ECM	-	52.593	35.942	
	+	55.059	38.408	1.0686105
	background	16.651		

ECM	-	95.846	80.52	0.8276968
	+	112.608	97.282	
	background	15.326		

	1	0.75749
	1	1.068611
	1	0.827697
	1	1.077838
AVE	1	0.932909
SE	0	0.08229

number of liver meta

#	Control	#	Digoxin
1	1	1	0
2	0	2	0
3	0	3	0
4	1	4	1
5	2	5	0
6	2	6	0
7	0	7	0
8	1	8	0
9	2	9	1
		10	0

	average	SD	SE	fold
Control	1	0.8660254	0.2886751	0.6667
Digoxin	0.2	0.421637	0.1333333	0.2

ｔ test

ｐ＝0.0185

Cont	Digo
0	0
0	0
197.3415	0
5050.05	264.62
4325.91	0
1568.04	0
0	0
973.08	0
438.59	0
	713.62

Luminescence level

	average	SD	SE
Control	1394.779056	1836.203	612.0675726
Digoxin	97.824	219.905	69.54005314

mouse GC model

Control0/Digo1	body weught
1	23.01
0	19.93
0	18
0	22.58
1	24.45
1	25.93
1	18.93
0	18.95
0	17.88
1	18.48
0	18.94
1	26.78
1	22.24
0	20.17
0	22.31
0	21.45
1	19.05
1	16.89
1	22.16

	average	SD	SE
Control	20.02333	1.762257	0.587419
Digoxin	21.792	3.359834	1.062473

t test p=0.176

GC tumor weight
mouse GC model

#	Control0/Digo1	weight
1	1	0.59
2	0	0.59
3	0	0.53
4	0	0.64
5	1	0.5
6	1	0.69
7	1	0.64
8	0	0.57
9	0	0.5
10	1	0.51
11	0	0.55
12	1	0.81
13	1	0.53
14	0	0.55
15	0	0.71
16	0	0.68
17	1	0.77
18	1	0.69
19	1	0.9

	average	SD	SE
Control	0.5911111	0.0709656	0.0236552
Digoxin	0.663	0.1350761	0.0427148

CTC number	Score
1	2
2	2
3	2
4	1
5	1
6	1
7	1
8	0
9	0
10	0
11	2
12	2
13	2
14	2
15	2
16	2
17	1
18	0
19	2
20	1
21	2
22	2
23	2
24	0
25	0
26	2
27	1
28	1
29	2
30	0

	average	SD	SE
PM translocation score	1.266667	0.81377	0.148573

CTC/ml

GC mouse model

#	Control	#	Digoxin
1	18.75	1	1.4
2	11.66	2	8.57
3	25	3	0
4	44	4	11.25
5	23.33	5	7.14
6	30	6	2.86
7	6	7	3.33
8	68	8	0
9	55	9	5.556
		10	36

	average	SD	SE
Control	31.304444	19.316351	6.4387837
Digoxin	7.6106	10.098873	3.193544

p = 0.0051

No	cohort		tumor weight	tumor length	CTC/ml	fold
1	Cont	0	1.73	21	122.5	1.854
2	Cont	0	2.42	17.14	78.7	1.453
5	Digo	1	2.21	18.63	2.5	1.009
6	Cont	0	1.34	17.37	0	1.102
8	Cont	0	1.88	17.49	10	1.058
9	Digo	1	2.46	19.18	3.3	1.441
10	Digo	1	1.69	16.48	7.5	1.163
11	Cont	0	2.55	19.79	47.5	1.388
12	Digo	1	2.98	18.99	0	1.377
13	Cont	0	2.65	19.01	50	1.317
14	Digo	1	2.1	16.04	1.25	1.078
15	Cont	0	2.81	20.95	15	1.122
16	Digo	1	2.86	19.23	5	0.856

Control	CTC/ml	weight	fold	Digoxin	CTC/ml	weight	fold
1.73	122.5	1.73	1.854	2.21	2.5	2.21	1.009
2.42	78.7	2.42	1.453	2.46	3.3	2.46	1.441
1.34	0	1.34	1.102	1.69	7.5	1.69	1.163
1.88	10	1.88	1.058	2.98	0	2.98	1.377
2.55	47.5	2.55	1.388	2.1	1.25	2.1	1.078
2.65	50	2.65	1.317	2.86	5	2.86	0.856
2.81	15	2.81	1.122				

**CTC/ml**	average	SD		SE
Control	46.24286	40.2001726		15.19423705
Digoxin	3.258333	2.45805083		1.00349505

**tumor weight**	average	SD		SE
Control	2.197143	0.50826636	Control	0.192106627
Digoxin	2.383333	0.44349621	Digoxin	0.18105657
